# A Noninvasive Assistant System in Diagnosis of Lumbar Disc Herniation

**DOI:** 10.1155/2020/6320126

**Published:** 2020-04-03

**Authors:** Miodrag Peulić, Miloš Joković, Tijana Šušteršič, Aleksandar Peulić

**Affiliations:** ^1^University of Kragujevac, Faculty of Medical Sciences, Serbia; ^2^Clinical Center Kragujevac, Serbia; ^3^University of Belgrade, Faculty of Medicine, Serbia; ^4^Clinic for Neurosurgery, Clinical Center of Serbia, Belgrade, Serbia; ^5^University of Kragujevac, Faculty of Engineering, Serbia; ^6^University of Belgrade, Serbia

## Abstract

The purpose of this study is the application of pressure sensors in diagnostics and evaluation of the accuracy diagnostics of lumbar disc herniation at levels L4/L5 and L5/S1 using the aforementioned platform. The motivation behind the idea to apply the pressure measurement platform is the fact that the motor weakness of plantar and dorsal flexia of the feet is one of the absolute indications for the operative treatment of patients with lumbar disc herniation at the indicated levels. In patients, MRI diagnosis of the lumbosacral spine served as the ground truth in the diagnosis of herniation at L4/L5 and L5/S1 levels. The inclusive criteria for the study were the proven muscle weakness based on manual muscle tests performed prior to surgery, after seven days of surgery and after physical therapy. The results obtained with the manual muscular test were compared with the results obtained using our platform. The study included 33 patients who met the inclusion criteria. The results of the measurements indicate that the application of our platform with pressure sensors has the same sensitivity diagnostics as a manual muscle test, when done preoperatively and postoperatively. After physical therapy, pressure sensors show statistically significantly better sensitivity compared to the clinical manual muscle test. The obtained results are encouraging in the sense that the pressure platform can be an additional diagnostic method for lumbar disc herniation detection and can indicate the effectiveness of operative treatment and physical therapy after operation. The main advantage of the system is the cost; the whole system with platform and sensors is not expensive.

## 1. Introduction

Lumbar pain syndrome is the second most frequent reason for reporting to a doctor. It is believed that fifteen percent of all absences from work come from pain in the back, and it dominates as a reason for sick leave in the population under the age of forty-five years [1]. In addition, besides being the undeniable and huge health problem, the lumbar disk herniation is also a socioeconomic problem that greatly burdens the health and social budget of states, due to allocation for medical expenses and payment for sick leave [2]. Discus hernia represents an intervertebral disk prolapse. The intervertebral disk provides stability in rest state and distributes the load of the spinal column on the move. Changes that lead to the occurrence of disc prolapse are desiccation, reduction of proteoglycan content, mucoid degeneration, and fibrosing tissue uptake. Predisposing factors of lumbar pain are heavy physical tasks with lifting loads over twenty pounds, especially professional use of heavy tools. There is considerable inconsistency in the literature, in the terminology and synonyms used in the description of the discus hernia [3]. Herniation is defined as localized prolapse of disk material beyond the boundaries of the intervertebral disc space. The hierarchy can be anterior, vertical, and rear. It is considered focal if it is less than 25% of the intervertebral disc circulation. By widespread prolapse, we consider the migration of disk material from 25% to 50% of the circulation of the space. Under the discontinuity, we mean the prolapse of the disc fragment whose width of the “neck” is no less than any other dimension of the fragment, as opposed to extrusion. By sequestration, we mean the presence of free fragments of the intervertebral disk in the spinal canal, while migration implies that the extruded material is far from the level of herniation, either as a sequester or in continuity. About 65% of migrations are ascendant. There is also the possibility of transdural migration of the discus.

Approximately 75% of the lumbar flexion and extensions are performed in the lumbosacral joint at the level L5/S1, 20% at the level of L4/L5, and the remaining 5% at the upper lumbar levels. This is why the lumbar disc herniation is localized in 90% of cases to the lower two levels, with those at the L5/S1 level being twice as common as the adjacent upper level. In the physical finding of patients with lumbar disc hernias, antalgic scoliosis, leg pain, motor weakness, sensory outbreaks, and hyporeflexia are recorded. There are positive root stretch tests and intensification of palpation pain in the area of the biliary gland. Lumbar disc herniation may happen with the front or rear migration of the pulmonary nucleus. Recent lumbar disc hernias can be dorsomedic occurring in 2% of all hernias or, significantly more often, dorsolateral with discoradicular contact, root compression, and radical pain. Horizontal disc prolapse is more common due to the anatomical characteristics of the last longitudinal ligament whose fibers are thinner in the lateral parts. Because the nerve root in the lumbar region goes below the pedicle of the same name and the intervertebral space is far below the pedicle, the prolonged disc compresses the nerve root that exits the intervertebral foramen of one level.

In the L4/L5 unilateral disc herniation, the disc is compromised at the root of L5 with a characteristic sensitivity decrease on the front lateral side of the lower leg, the upper side of the foot, and the sensation of sensitivity towards the foot of the foot. Motor weakness affects m. tibialis anterior and m. extensor hallucis due to which the patient has a weakness while standing on the heel. In the unilateral disc herniation at the L5/S1 level, the root S1 is compromised, with a characteristic sensitivity decrease on the back of the lower leg, an outer part of the heel, and a sensation of sensitivity toward the small finger of the foot. The fatality affects the peroneal musculature and m. triceps of the surae, which is impaired with standing on the forefeet (fingers on toes).

The weakness while standing on the heel and forefeet is determined by a manual muscle test. During testing, an appropriate technique must be applied to ensure valid results. Clothes are removed to clearly see the contraction of the muscle. The activity of muscle agonistic groups should be excluded.

The most commonly accepted method of evaluating muscle strength is the Medical Research Council Manual Muscle Testing scale. Strength of the muscles is tested, initially, by performing the test horizontally, therefore eliminating the effects of gravity. Muscles should first be tested with gravity eliminated by positioning the patient, so that muscle contraction is perpendicular to gravity, such as along an examining table or bed. If the patient is unable to engage the muscle even after the gravity has been eliminated, the examiner puts his hand on the muscles and asks the patient to engage the muscle. This allows the examiner to feel muscular contraction even if it is not visible to the examiner. This sets the difference between scores of 0 and 1. When the patient successfully engages the musculature by excluding the gravity force, we examine the strength of the muscles against the force of gravity. If the patient successfully performs a movement against the force of gravity, the examiner increases the force of movement resistance to the maximum, which definitely assesses the degree of muscle strength of the examined muscle or muscle group. The manual test of muscular strength in patients is easy to perform and does not require any specialized equipment, in which we consider its basic advantage. Despite these advantages, this technique also has its limitations. Scoring is subjective and based on the perception of the examiner. There is also a significant variability among patients because some respondents are stronger than others. The test does not take into account the musculoskeletal disorders that can affect the test, such as tendinopathy or arthritis. The test depends on the motivation of the patient, which may be insufficient in some patients, because of pain, misunderstanding of instructions, psychological reasons, or secondary reasons.

### 1.1. Muscle Strength Grading

Muscle strength is quantified based on a standard scale used to determine the degree of motor weakness which, at the moment, represents the “gold standard” [4]:

0 = absence of contraction

1 = minimal contraction that does not cause movement

2 = movement which exists but can not be performed against the force of gravity

3 = movement which cannot overcome the resistance of the examiner

4 = movement which overcomes resistance of the examiner to a certain degree

5 = normal muscular strength

Obviously, there is an insufficiently clear range between 3 and 5, making the determination subjective. Some examiners expand the scale of 5 degrees to 9 degrees by adding the symbols “+” and “-” when the force appears to be between the defined gradients. Hence, the scale is quite arbitrary and insufficiently precise [5, 6].

Radiological diagnostic methods used in the diagnosis of lumbar disc hernias are computerized tomography, nuclear magnetic resonance, myelography, and rarely discography [7–9].

### 1.2. Treatment Options for Lumbar Disc Herniation

Initial treatment of a confirmed disc hernia is conservative, except in patients with an absolute indication for operative neurosurgical treatment, under which the presence of sy. caudae equinae is meant, a progressive neurological deficit and expressive motor deficit [10–12]. Relative indications for operative treatment are intense pain that can not be dealt with conservatively. Approximately 5-10% of patients with persistent pain in the lower back require operative treatment [13].

### 1.3. Platform with Foot Pressure Sensors

**Figure 1:**
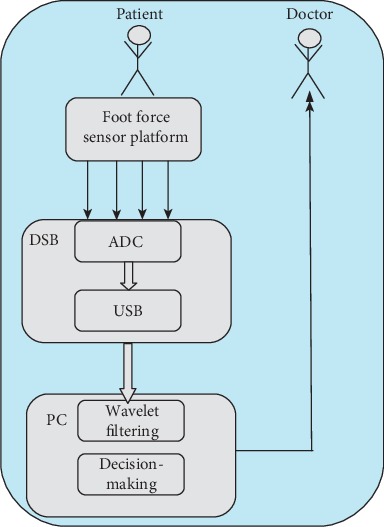
Blok diagram of the measurement system.

The hardware part consists of an electronic printed acquisition card, and the software part includes the software code on the microcontroller and the software code for the user application that runs on the PC. For those purposes, we introduce the measurement system consisting of two identical plate dimensions 32 × 30 cm. An unavoidable component of the development system is the microcontroller used to collect data (Figure 1).

**Figure 2:**
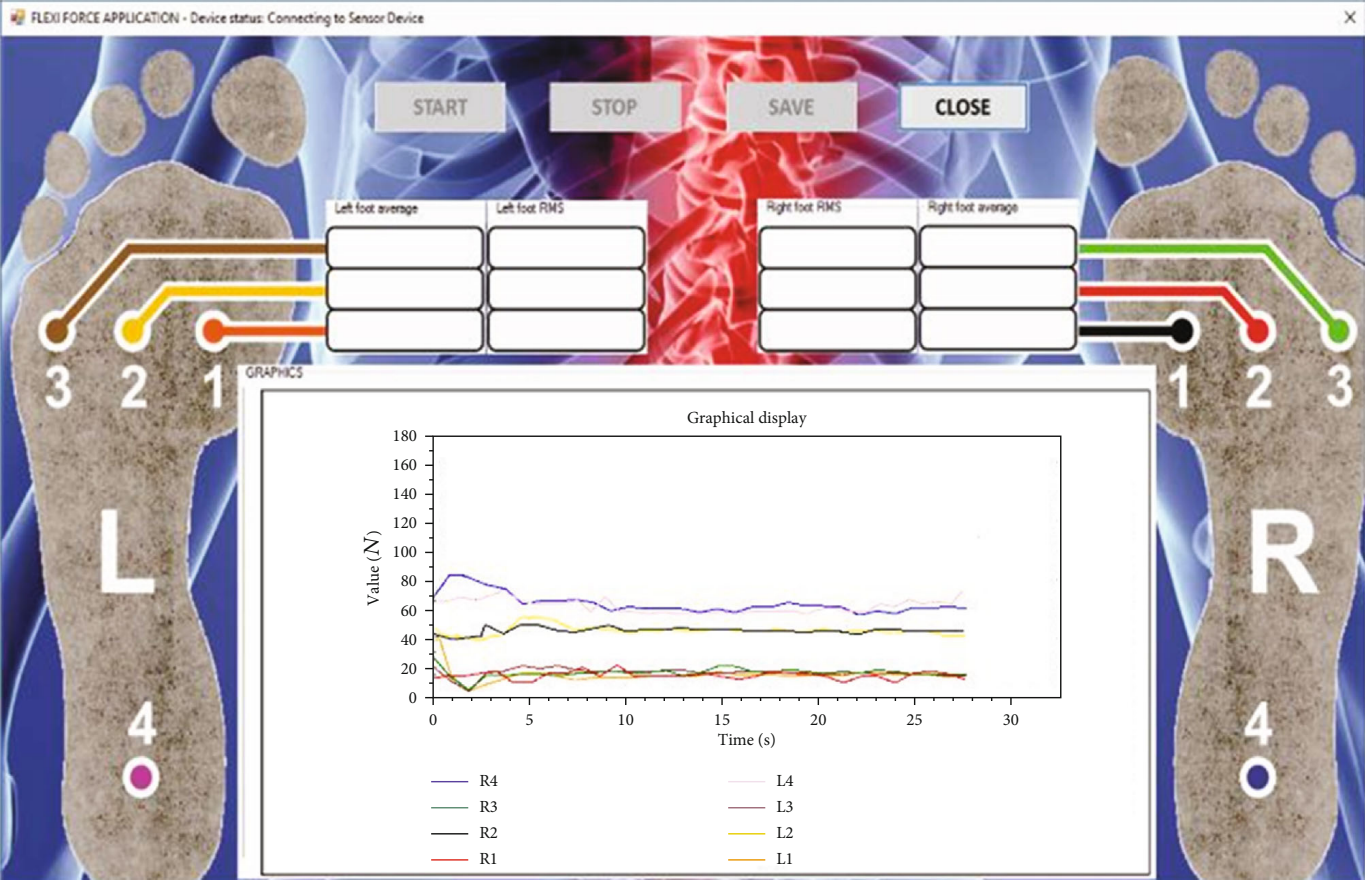
Main user measuring plate application window.

Portable systems are becoming part of everyday clinical practice [14, 15]. Systems for the surface distribution of foot pressure were applied in patients with diabetes and rheumatoid arthritis, as well as in the development of sports equipment and injury prevention [16–19]. However, they are never applied in diagnostics for disc hernia and classification of the level of herniated disc. An application was developed in the Visual Studio C# software package to measure and display the measurement results on a computer (Figure 2). The measurement accuracy is defined by the calibration guaranteed by the manufacturer of the sensor and the accuracy of the system by calibration using the etalon of weight.

**Figure 3:**
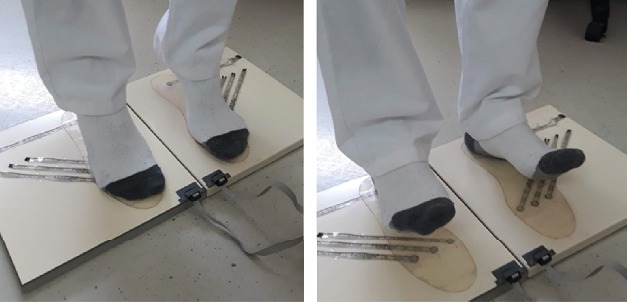
The process of measurement using the measurement platform.

The plates are made of a hard-pressed wood coated with a millimeter-thick layer of cant tape. In order to make the substrate flat and smooth, the plates are coated with a lacquer, reducing the possibility of surface roughness to a minimum. On both plates, four flexi force sensors, which can measure up to 440 N, are symmetrically arranged [20]. The sensors are placed according to a specific arrangement that corresponds to the human foot, four sensors on each measuring plate (Figure 3). The sensors are labeled as L1-4 for the left foot and R1-4 for the right foot (Figure 2). A specific sensor position was chosen to represent characteristic points of the human foot. Sensors were mounted under the first, second, and fourth metatarsal heads as well as the heel, because these characteristic points are in accordance with the manual diagnosis procedure used during the manual muscle testing. Additionally, we have also tested the same system with up to 8 sensors per foot, and the results were the same, leading to the conclusion that 4 sensors were sufficient to catch the investigated phenomena of muscle weakness on the feet of the patients with L4/L5 and L5/S1 disc hernia. In order to protect the sensor system from physical damage, the testing surface is coated with a 1 mm thick rubber layer. Sensors are connected with conductors at the ends of the panels containing CFC connectors on the platform of the measurement system, which is then connected with the acquisition card via the stripline (flat) cable.

Research design is as follows: the patient used the constructed platform to record the raw signals via four sensors per foot, which were further processed in order to make a final decision about the disc hernia on the level of L4/L5 and L5/S1 on the left or right side. Explanations of the details of the decision system are given in the Material and Methods section.

## 2. Material and Methods

The study included patients older than eighteen years who suffer from lumbar disc herniation at L4/L5 and L5/S1 levels, confirmed by nuclear magnetic resonance, in whom an absolute indication for operative neurosurgical treatment is set based on the presence of motor deficits. Patients with disc hernia at the level of L3/L4 were not included in this study since there is no consistency in the relationship between the muscle weakness in the foot and this diagnosis. When the disc herniation is present on either of the levels of L4-L5 or L5-S1, as a result, the nerves suffer pressure on that level. This causes the muscle weakness on the corresponding part of the left or right foot because the innervation of the muscles and skin area on the toes originates from the nerves between L5 and S1 discs in the spine, while innervation of the heels comes from the nerves that originate between L4 and L5 discs in the spine. This represents the foundation of the application of sensors in objective measurement of muscle weakness on different areas of feet.

Only patients who had surgery of lumbar disc herniation for the first time are included in the study. Only patients with no other spinal problems but disc hernia disease on the level of L4/L5 and L5/S1 are included in the dataset, meaning patients with disease affecting multiple discs, spinal stenosis, cauda equina syndrome, spondylolisthesis, neurogenic claudication, or previous spinal surgery were excluded from the dataset. Patients with pathologies of the lumbar spine, including tumors, infections, inflammatory spondylarthropathies, fractures, Paget's disease, severe osteoporosis, diabetes, and pregnancy were also excluded from the dataset. The criteria for exclusion from the study were also incomplete data in the history of the disease and other medical records, as well as the existence of joint injuries or diseases that may affect the test results.

Prior to force measurement, subjects were introduced to the measurement system and tried the procedure once before the real testing procedure. Education time lasted only a couple of minutes, as it is very simple and similar to weight measurement. The testing procedure is divided into three parts:

1. The subject is standing on both feet normally (like weight measurement)
2. The subject is standing on both feet, but the weight is transferred to the forefeet (term forefoot here is used for leaning towards feet metatarsal heads)
3. The subject is standing on both feet, but the weight is transferred to the heels

One continuous recording of the three submeasurements was performed, and how to perform the testing procedure was explained and demonstrated by the physician to each patient. The patients were instructed not to make any sudden movements during the measurement (i.e., bending the body sideways or uncontrolled movements of the hands). Measurement procedure time was not fixed, but it was done until the three characteristic segments (standing on both feet normally, standing on forefeet, and standing on heels) could be recorded properly. The reason behind is that sometimes patients were unstable and made sudden movements or even kept falling. Therefore, recording was repeated until the patients could find the balance and the three submeasurements are recorded. However, in each patient, the recording lasted not more than 5 minutes. The sampling time was 10 ms.

In order to improve the quality of raw measured signal, wavelet transformation is performed before analyzing. Because Fourier transformation is not suitable for processing and analyzing of stationary signals, for analyzing these signals, wavelet transformation is used. Wavelets are the functions which satisfy some conditions such as oscillatory nature. Wavelet transformation provides analysis of signals with different frequencies by using different resolutions, i.e., usage of the windows of different widths during signal analysis. This means that each spectral component is not equally analyzed, which is the case if fast Fourier transformation is used. Therefore, wavelet transformation is designed to give good time and bad frequent resolution on the signal part with high frequency and good frequent and bad time resolution on the signal part with low frequency. This approach is especially sensitive when signal contains components with high frequencies and short duration and low frequency and long duration, which is case with medical signals.

After that, based on the doctor's experience and interpretation of scoring on the Oswestry Low Back Pain Disability Questionnaire [21], we have adopted score recommendations following a threshold for muscle weakness. We calculate the mean value for each sensor and normalize the values to fall into the range 0-1. Based on that, we obtain one attribute per sensor. We further average three values for the three sensors on forefeet, leading to the two final attributes per foot (denoted as *toes_left* and *heel_left* for the left foot and *toes_right* and *heel_ right* for the right foot). This leads to further definitions:

- If the attribute value is less than 0.3, we consider that there is a very strong muscle weakness pointing to the level of discus hernia (further denoted as VERY_LOW, meaning very low stability)
- If the attribute value is between 0.3 and 0.7, we consider there is some muscle weakness pointing to the level of discus hernia (further denoted as LOW, meaning low stability)
- If the attribute value is greater than 0.7, we consider there are normal stability and no indications of discus hernia (further denoted as NORMAL)

Finally, decision is made based on the expert knowledge, which in our case is defined by the medical doctor. We have defined 42 rules that describe the process of diagnosing the level of disc hernia. Examples of some rules are as follows:

- If *toes_left* is VERY_LOW and *toes_right* is NORMAL and *heel_left* is NORMAL and *heel_right* is NORMAL, then *diagnosis* will be L5/S1 ON THE LEFT SIDE
- If *toes_left* is NORMAL and *toes_right* is VERY_LOW and *heel_left* is NORMAL and *heel_right* is NORMAL, then *diagnosis* will be L5/S1 ON THE RIGHT SIDE
- If *toes_left* is NORMAL and *toes_right* is NORMAL and *heel_left* is LOW and *heel_right* is NORMAL, then *diagnosis* will be L4/L5 ON THE LEFT SIDE
- If *toes_left* is NORMAL and *toes_right* is NORMAL and *heel_left* is NORMAL and *heel_right* is LOW, then *diagnosis* will be L4/L5 ON THE RIGHT SIDE

Demographic data for the study was obtained from the patient history disease: age, gender, clinical status, neurological deficit, and data on the results of the lumbosacral region recorded by nuclear magnetic resonance. For each patient, a type of operative approach was also noted, that is, classical discectomy or microdiscectomy.

After operative treatment, all patients were sent to physical therapy. After the completed physical therapy, a control check was scheduled, where the manual muscle tests and measurements using the pressure sensing platforms were repeated. Manual muscle tests were performed by two neurosurgeons from the Center for Neurosurgery, KC Kragujevac. The process of measurement on the original platforms was carried out by a research neurosurgeon during the stay of patients in the department and on the control examination, after the conducted physical therapy.

Preoperatively, a manual muscle test and initial measurement using surface pressure distribution platforms were performed. After the operation, on the seventh postoperative day, a manual muscle test and a repeated measurement using platforms were done. For all measurements, our designed platform for measuring the pressure distribution of the foot was used.

## 3. Statistical Analysis

Statistical analysis was carried out to compare the “gold standard” (clinical test of motor weakness) with our constructed platform. Continuous data were reported with median and ranges. For later analysis, patients were classified into the type of obesity based on BMI as follows:

1. <18.5: underweight
2. 18.5–24.9: normal weight
3. 25.0–29.9: overweight
4. 30.0–34.9: class I obesity
5. 35.0–39.9: class II obesity
6. ≥40.0: class III obesity

The comparison of continuous data was performed with Student's *t*-test and Wilcoxon's test. For the comparison of categorical data, the chi-square test was performed. Spearman's correlation analysis was used to examine the relationship between values measured by MMT and plates. Logistic regression was used to explain the relationship between two diagnostic tests. The ROC curve was used for calculating the cut-off value of the performed diagnostic test and to determinate specificity and sensitivity. The level of statistical significance in all analyses is set to 0.05. All calculations were performed using commercial program SPSS, version 20.

## 4. Results

The total number of patients who were operated at the Center for Neurosurgery KC Kragujevac, in the period from December 2014 to February 2018, was 203, out of which 33 satisfied the inclusion criteria for the study. Seventeen of them (51.5%) were men, and sixteen (48.5%) were women (*p* = 0.862). On the L4/L5 level, disc herniation had 14 patients and 19 patients had herniation on the L5/S1 level; 16 patients had left sided disc herniation, and 17 patients had right sided (*p* = 0.356). Microdiscectomy was performed at 20 (60.6%) and classical discectomy at 13 (39.4%) patients (*p* = 0.223) (Table 1).

The mean (range) strength value measured by a platform preoperatively was 6.382 (0.113-15.566), postoperatively was 11.613 (0.254-19.530), and after physical therapy was 12.985 (1.517-20.533). Manual muscle test values before operation, after operation, and after physical therapy were 3 (1-4), 4 (2-5), and 4 (3-5), retrospectively.

When we compared strength results measured by plates, values after operation were significantly higher compared to values before operation (*p* < 0.01) and values after physical therapy were significantly better compared to values after operation (*p* = 0.007). Strength results measured by MMT were significantly better after operation compared to preoperative results (*p* < 0.01), and values after physical therapy were significantly higher than postoperative values (*p* = 0.018).

**Figure 4:**
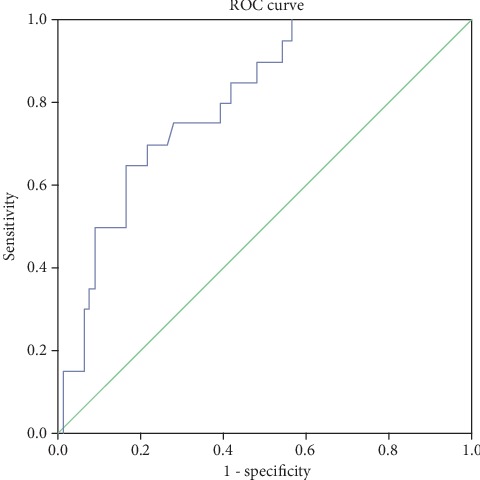
ROC curve for the platform testing.

The correlation between results of MMT testing and pressure sensors was significant (*r* = 0.606, *p* < 0.01). Logistic regression was used to determine the relation between results which are obtained from MMT testing as a gold standard and from testing with plates. The whole model was statistically significant (*c*^2^ = 19.773, *p* < 0.01). This result is expected as it was explained in the Material and Methods section; innervation of the muscles and skin area on the toes originates from the nerves on level L5 and S1 discs in the spine, while innervation of the heels is done via nerves that originate between L4 and L5 discs in the spine [1, 2]. This is shown on dermatome maps for the lower limbs. Physicians use this medical background in the examination of the level of discus hernia during muscle tests, performing a neurological exam by pressing the patient toes and heels. In this way, physicians are trying to determine muscle weakness on the toes/heels of the subject. However, the conclusions about muscle weakness that are made via these tests are subjective and could be biased. That was our main motivation to build the platform for the objective measurement of muscle weakness and disc hernia diagnostics. Values computed by plate testing showed predictive importance for strength recovery (AUC = 0.802, *p* < 0.01) (Figure 4), and values which are higher than 12.712 can be considered indicators of normal muscle strength with a specificity of 0.722 and a sensitivity of 0.750.

The correlation coefficient between the gender and level of disc herniation was 0.279, and that between obesity type and level of disc herniation was 0.037. This shows very weak or almost nonexisting correlation, which was not statistically significant. There was also no statistical significance in gender or obesity type related to the specific level of disc herniation. We can conclude that there was no strong relationship between any of these variables, meaning there was no systematic error in diagnosing the level of disc herniation, in the sense that neither of the genders was biased for one level of herniation. This also means that there is no preference in the obesity type and certain level of disc herniation.

## 5. Discussion

Lumbar pain due to disc herniation is one of the most frequent causes of morbidity and absence from work. Because of that, it has great health and socioeconomic importance. Evaluation after operative treatment has great importance in a patient's recovery. In most cases, evaluation is based on manual muscle testing (MMT). One of the most imperfections of MMT is the great level of subjective evaluation of the patent's strength recovery. We wanted to construct a system which is more objective. In this work, we have compared the plate system with MMT as a gold standard from muscle strength examination. From 203 operated patients, we examined 33 patients who satisfied the inclusion criteria. In our study, we did not find a statistically significant difference between the numbers of men and women. Also, there was no significant difference in the level and side of disc herniation. We did not find a statistically significant difference in the type of operation (microdiscectomy and classic discectomy).

Median muscle strength measured by MMT postoperatively showed improvement compared to the value before operation, and median MMT after physical therapy was the same as postoperative values (although according to ranges 3-5 after physical therapy and 2-5 after operation) so we can notice improvement in the muscle strength. We obtained a statistically significant difference between preoperative and postoperative values after physical therapy.

Muscle strength measured by plates was getting better and better after operation and physical therapy, and we showed a significant statistical difference between all groups of patients.

We showed significant correlation between these two tests. Also, we calculated the cut-off value which can be used to determine normal muscle strength.

The main limitation of this study was that foot size was not taken into account. However, we have obtained also small correlation (correlation coefficient was 0.215) between the gender and correctly (and incorrectly) diagnosed level of disc herniation by the platform. Similarly, correlation between obesity type and correctly (and incorrectly) diagnosed level of disc herniation by the platform was 0.156. Because of this, we have strong implications that our system is able to diagnose correctly disc herniation independently of the foot size. However, in order to confirm these implications, foot size will be considered in future research testing. This will be done in order to adjust the current platform to the noninvasive assistant system in the clinical system for daily use.

We have also shown that the system is able to successfully distinguish healthy individuals from the patients with discus hernia. The main aim of our platform is to provide a low-cost, noninvasive system that can meet the need to have the fast and accurate diagnosis of disc hernia, saving a clinician's and a patient's time and reducing time in waiting in the queues for MRI. So far patients with mixed sensory injury, index relation to pain, motor, and sensory or some border cases were not tested, as this was the first time that someone introduced the platform for measurement in order to diagnose discus hernia. First, we wanted to prove that the system can detect patients with clear diagnosis (no mixed injury or more than one discus hernia present) and then extend its use to further applications and adjustments.

In order to adjust the developed system for the real clinical use, we aim to investigate further to improve accuracy, including foot size, mixed sensory injury, and some border cases in analysis, and implement the whole system to include measuring, processing, and decision-making in real time, on-site, whether in hospitals or in homes.

## 6. Conclusion

The measurement of muscle strength showed significant improvement after operation and after physical therapy. Also, values which are obtained by measuring with our platform are in correlation with MMT values. Measuring by plates is more precise, and its importance lies in its objectivity because measuring does not depend on a person who performs examination. One more contribution of plates is obtaining the value that can be used as a reference value for determining normal muscle strength.

## Figures and Tables

**Table 1:** Number of patients per different categories that met the inclusion criteria.

	Number of patients
Gender	
Male	17
Female	16
Herniation level	
L4/L5	14
L5/S1	19
Herniation side	
Left	16
Right	17
Surgery type	
Classic discectomy	13
Microdiscectomy	20

## Data Availability

The data used to support the findings of this manuscript are restricted by the Center for Neurosurgery KC Kragujevac in order to protect patient privacy and avoid legal and ethical risks. Data are available from the Center for Neurosurgery KC Kragujevac for researchers who meet the criteria for access to confidential data and can be obtained by contacting the first author.
